# Is coronary artery tortuosity a precursor of atherosclerosis and/or left ventricular diastolic dysfunction?

**DOI:** 10.1186/s43044-021-00192-3

**Published:** 2021-07-28

**Authors:** Zainab Atiyah Dakhil

**Affiliations:** Al-Kindy College of Medicine, University of Baghdad, Baghdad, Iraq

To the Editor,

RE: “Coronary tortuosity relation with carotid intima-media thickness, coronary artery disease risk factors, and diastolic dysfunction: is it a marker of early atherosclerosis?”, March 31, 2021.

We read with interest the thoughtful paper by Ahmed Alamragy et al. [1] that highlighted the association of coronary artery tortuosity (CAT) with increased carotid intima-media thickness (CIMT), hypertension, hyperlipidemia, left ventricular diastolic dysfunction (DD), and coronary artery atherosclerosis, so this study contributes well to the existing literature especially if we consider how common is this so-called benign coronary phenotype and how limited data are there regarding its significance. Nevertheless, there are certain issues that we would like to raise regarding this study.

This study referred only to tortuous main coronary arteries without mentioning if sizable side branches’ tortuosity was excluded or not from the control group; side branch tortuosity has been reported as negative predictor of atherosclerosis [2], and we think it might be important to at least mention if they were considered especially if they were large side branches that irrigate large area of myocardium.

The researchers did well with choosing age- and gender-matched control patients because of the debate of the impact of these factors on CAT; however, one of the main findings of this study is the diastolic dysfunction that was observed more in the CAT group, yet the researchers did not exclude other causes of DD from the CAT group like hypertension, obesity, and thyroid diseases [3, 4], and this might remarkably affect the results especially if we consider that the study showed that 86.7% of the CAT group vs. 30% of the control group were hypertensive which might explain the higher prevalence of DD in the CAT group. Despite that the researchers excluded uncontrolled hypertension (which the study did not define), still hypertension is well recognized cause of DD that might impact the results. This is more crucial if we consider that no multiple regression analysis was done to assess the predictors of DD in this study which could better waive the impact of the case selection issue.

The main question of this study was to determine the predictors for CAT, yet the title did not clearly reflect this objective; however, the question was clearly answered despite the small study population.

It is also noteworthy to mention that coronary artery tortuosity can be part of systemic arterial tortuosity [5], so carotid artery tortuosity can be associated with CAT, which might explain the increased CIMT here rather than the direct association of this CIMT increase with CAT, as the atherosclerotic changes in tortuous arteries are reported to be due to local shear stress forces, so such possibility needs to be evaluated in future studies especially with the conflicting existing literature.

Interestingly, assessment of coronary artery calcium burden by CT coronary angiography is a surrogate for coronary atherosclerosis, and its relation with CAT is also a matter of debate [6, 7], so it can be further investigated in future studies.

Despite the raised issues, we believe that this study can open the door for future researchers to further explore the significance of coronary artery tortuosity.

## Data Availability

Not applicable.

## Abbreviations

CAT: Coronary artery tortuosity
CIMT: Carotid intima-media thickness
DD: Left ventricular diastolic dysfunction
